# Trends in incidence and mortality of tuberculosis in Japan: a population-based study, 1997–2016

**DOI:** 10.1017/S095026881800290X

**Published:** 2018-11-09

**Authors:** H. Hagiya, T. Koyama, Y. Zamami, Y. Minato, Y. Tatebe, N. Mikami, Y. Teratani, A. Ohshima, K. Shinomiya, Y. Kitamura, T. Sendo, S. Hinotsu, K. Tomono, M. R. Kano

**Affiliations:** 1 Division of Infection Control and Prevention, Osaka University Hospital, 2-15 Yamadaoka, Suita, Osaka 5650871, Japan; 2 Department of Pharmaceutical Biomedicine, Graduate School of Medicine, Dentistry, and Pharmaceutical Sciences, Okayama University, 1-1-1 Tsushima-Naka, Kita-ku, Okayama, 7008530, Japan; 3 Education and Research Center for Clinical Pharmacy, Graduate School of Medicine, Dentistry, and Pharmaceutical Sciences, Okayama University, 1-1-1 Tsushima-naka, Kita-ku, Okayama 7008530, Japan; 4 Department of Clinical Pharmacology and Therapeutics, Tokushima University Graduate School of Biomedical Sciences, 3-18-15 Kuramoto, Tokushima, 7708503, Japan; 5 Department of Pharmacy, Tokushima University Hospital, 2-50-1 Kuramoto, Tokushima, 7708503, Japan; 6 Department of Microbiology and Immunology, University of Minnesota Medical School, 689 23rd Avenue SE, Minneapolis, Minnesota, 55455, USA; 7 Department of Pharmacy, Okayama University Hospital, 2-5-1 Shikata-cho, Okayama 7008558, Japan; 8 Division of Pharmacy, Chiba University Hospital, 1-8-1 Inohana, Chuo-ku, Chiba 2608677, Japan; 9 Department of Toji Pharmacy, Smile Co., Ltd., 6-1-11 Syoko-center, Nishi-ku, Hiroshima, 7330833, Japan; 10 Department of Biostatistics and Clinical Epidemiology, Sapporo Medical University, South 1, West 17, Chuo-Ku, Sapporo, Hokkaido 0608556, Japan; 11 Department of Pharmaceutical Biomedicine, Graduate School of Interdisciplinary Science and Engineering in Health Systems, Okayama University, 1-1-1 Tsushima-Naka, Kita-ku, Okayama, 700-8530, Japan; 12 Department of Geriatric Medicine, University of Tokyo, 7-3-1 Hongo, Bunkyo-ku, Tokyo 1138655, Japan

**Keywords:** Incidence, mortality, trend analysis, tuberculosis

## Abstract

Japan is still a medium-burden tuberculosis (TB) country. We aimed to examine trends in newly notified active TB incidence and TB-related mortality in the last two decades in Japan. This is a population-based study using Japanese Vital Statistics and Japan Tuberculosis Surveillance from 1997 to 2016. We determined active TB incidence and mortality rates (per 100 000 population) by sex, age and disease categories. Joinpoint regression was applied to calculate the annual percentage change (APC) in age-adjusted mortality rates and to identify the years showing significant trend changes. Crude and age-adjusted incidence rates reduced from 33.9 to 13.9 and 37.3 to 11.3 per 100 000 population, respectively. Also, crude and age-adjusted mortality rates reduced from 2.2 to 1.5 and 2.8 to 1.0 per 100 000 population, respectively. Average APC in the incidence and mortality rates showed significant decline both in men (−6.2% and −5.4%, respectively) and women (−5.7% and −4.6%, respectively). Age-specific analysis demonstrated decreases in incidence and mortality rates for every age category, except for the incidence trend in the younger population. Although trends in active TB incidence and mortality rates in Japan have favourably decreased, the rate of decline is far from achieving TB elimination by 2035.

## Introduction

Tuberculosis (TB) continues to be one of the leading infectious causes of death globally. In 2016, the global incidence of all forms of TB was 10.4 million, leading to 1.3 million annual deaths worldwide [1]. Furthermore, the emergence of multidrug-resistant TB, caused by various gene mutations [2], is a growing global public health concern [3]. In the World Health Organization (WHO)’s End TB Strategy, the WHO proposed the reduction of TB incidence to <10 cases per 100 000 population by 2035 and less than one case per 100 000 population by 2050, and eventually for the elimination of the disease [4]. In the era of globalisation, there is no TB-free region and this contagious disease cannot be eliminated from any region without a global approach. The TB burden is a major public health issue predominantly in low- and middle-income countries. However, it continues to be a persistent problem in high-income countries, among various vulnerable populations in each society.

In high-income countries with low-TB incidence, these goals require additional efforts. First, it is important to recognise common risk factors for TB exposure. Older people are known to be a common population who develop active TB disease [5,6]. Changes in the population structure of industrialised countries, i.e. ageing societies, may accelerate the overall risk of TB exposure [5–7]. Other specific populations, such as homelessness [8] and immigrants from countries with a high prevalence of TB [9,10], may also possibly diffuse TB. Thus, we should note that contacts to these population would pose a risk of TB exposure in advanced countries. Second, it is also essential to improve access to high-quality TB care and address the underlying risk factors for TB progression [11]. Medical risk factors, such as HIV [12], increasing drug resistance [13] and non-communicable diseases, such as diabetes mellitus [14,15], alcohol abuse and smoking [16], can allow latent TB infection to progress to active diseases. Better management of these underlying conditions would also lead to suppression of TB disease.

Japan is still a TB middle-burden country, with a notification rate of 13.9 cases per 100 000 population in 2016 [17]. A previous study revealed that a high incidence rate among older people is a possible explanation for the TB prevalence in Japan [5]. TB incidence has been consistently higher among older individuals aged >80 years in Japan, with 49.8 and 78.1 per 100 000 population among adults aged 80–84 and 85–89 years, respectively [17]. Also, as with European regions, an increase in TB cases among foreigners might have influenced the slowing down of the decline in the trend of TB in Japan [18]. To achieve the global goals for TB elimination, prioritisation of key measures and target populations should be based on an epidemiological study in each country and region, guided by effective data analysis. In Japan, trends in the crude rate of TB incidence have been reported [17]; however, epidemiological trend analysis on nationwide TB incidence and mortality has not been fully evaluated.

In this article, we aimed to evaluate the incidence and mortality trends of TB in Japan during the last two decades. Furthermore, the differences in comparison with other countries, to inform intervention efforts towards meeting the Sustainable Development Goals to tackle TB by 2035, were discussed [19].

## Materials and methods

### Data source

This was a 20-year retrospective observational study covering data from 1997 to 2016. Data used to determine newly notified active TB incidence were obtained from the Tuberculosis Surveillance Center in Japan [20]. Japan introduced the first nationwide computerised TB surveillance system in 1987. TB is a notifiable disease, and public health centres are responsible for the collection and entry of the data from notified patients into the system. The data are summarised every month and annually, and are made publicly available online [20]. Data on the number of TB-associated deaths by sex, age and TB categories (respiratory TB, nervous system TB and other TB) were obtained from the vital statistics collected by the Japanese Ministry of Health, Labor, and Welfare [21]. In Japan, TB is defined as a Category II Infectious Disease by the Act on the Prevention of Infectious Diseases and Medical Care for Patients with Infectious Diseases (the Infectious Diseases Control Law) [22]. Physicians who diagnose patients with TB disease are required to report the case to the National Epidemiologic Surveillance of Infectious Disease (NESID). Thus, patients with TB diseases are well diagnosed and reported throughout the entire country. The surveillance system data included information on the patient's sex, age and diagnosis of TB diseases. Age groups were categorised as: 0–24, 25–44, 45–64 and ⩾65 years. However, data on the mortality rate among people aged ⩽24 years were not included in the subgroup analysis because of the limited number of deaths in this age group. To improve comparability, we adopted the direct standardisation method, and calculated the age-adjusted rates (AARs) of incidence and mortality, based on the European standard population [23] as a standard of industrialised countries, using 5-year age groups. Ethics approval was waived by the institutional review board in Okayama University Hospital, since the data used in the present study were anonymised and open to the public.

Definitions of TB-related death were based on the International Statistical Classification of Diseases and Related Health Problems coding system (10th edition), according to previous literature [24–26]. Accordingly, TB diseases were defined as follows: respiratory TB (A15 and A16), TB of the nervous system (A17), TB of other organs (A18) and miliary TB (A19).

### Statistical analyses and data processing

We calculated crude and AARs of active TB diseases and TB-related death. Age adjustment is one of the key methods to control for different age distributions among populations, or over time. To estimate trends in the AAR, the Joinpoint regression model was applied for sex, age and TB categories using the Joinpoint Regression Program, version 4.5.0.1, June 2017 (Statistical Research and Applications Branch, National Cancer Institute) [27], which was applied in previous literature [24]. We used Joinpoint regression analysis to identify years when significant trend changes occurred in the linear slope of the temporal trend. This analysis has two strengths. The analysis identifies the year when significant trend changes occurred and estimates the magnitude of the increase or decrease in each linear slope by calculating the annual percentage change (APC). Also, the average annual percentage change (AAPC) in the entire period and the current 5-year period was estimated. Data processing and aggregation were performed using Microsoft Access^®^ 2013 (Microsoft Corporation, Redmond, WA, USA).

## Results

### Level changes in incidence and mortality rate

**Fig. 1.**
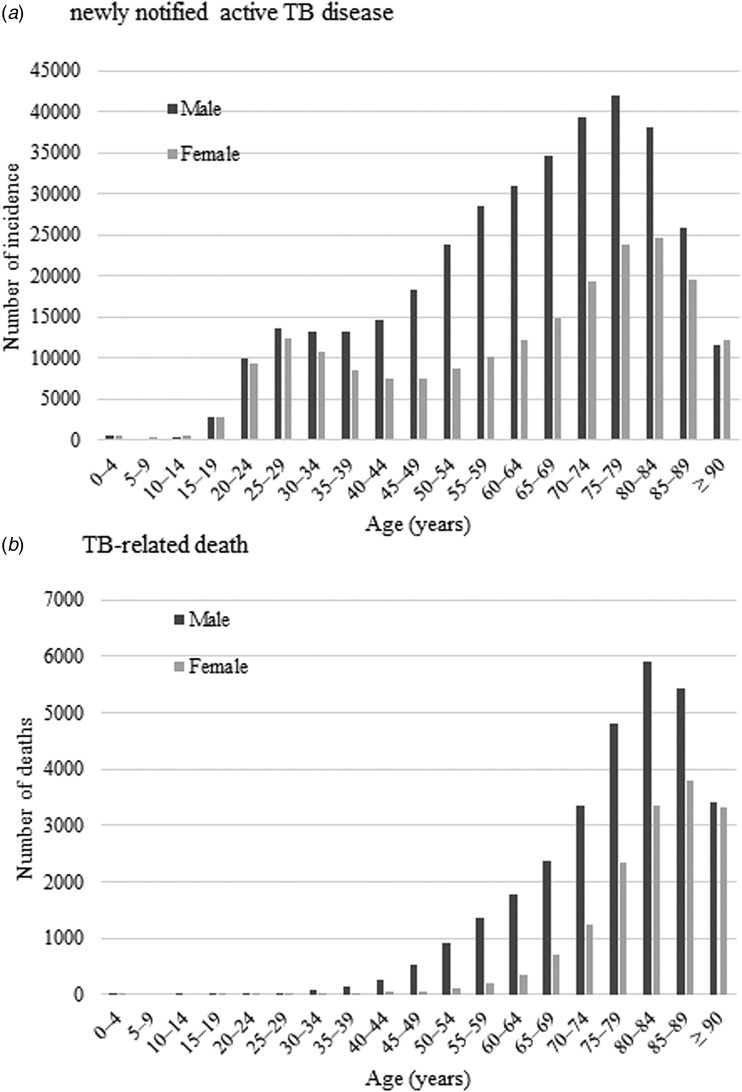
Age–sex distribution of newly notified active tuberculosis incidence (a) and tuberculosis-related deaths (b) during 1997–2016.

During the study period, the incidence of TB chronologically declined from 1997 (42 715 cases overall; with 27 384 in males and 15 331 in females) to 2016 (17 625 cases overall; with 10 594 in males and 7031 in females) (Table 1 and Fig. 1). The age-adjusted incidence rate per 100 000 population in males (15.8) was two times higher than that in females (8.0) in 2016. Similarly, the number of TB deaths reduced from 1997 (2742 cases overall; 1955 in males and 787 in females) to 2016 (1892 cases overall; 1133 in males and 759 in females). Between 1997 and 2016, the crude and age-adjusted mortality rates per 100 000 population decreased from 2.2 to 1.5 and 2.8 to 1.0, respectively. Between sexes, the age-adjusted mortality rate per 100 000 population among males (1.7) was three times higher than that in females (0.6). Among Japanese, both TB incidence and death involved men more frequently than women in most age groups, including the older population. Although this decreasing trend was preferable, the incidence of TB was far from the End TB goals to achieve by 2035.

**Table 1.** Newly notified active tuberculosis (TB) incidence and TB-related mortality rates per 100 000 persons during 1997–2016

Year	Incidence rate per 100 000			Mortality rate per 100 000		
	No. of incidence, thousands	Crude rate	AAR	No. of TB death	Crude rate	AAR
1997	42.7	33.9	37.3	2742	2.2	2.8
1998	41.0	32.4	35.1	2795	2.2	2.7
1999	43.8	34.6	37.1	2935	2.3	2.8
2000	39.4	31.0	32.7	2656	2.1	2.5
2001	35.5	27.9	28.9	2491	2.0	2.2
2002	32.8	25.8	26.2	2317	1.8	2.0
2003	31.6	24.8	24.9	2337	1.8	2.0
2004	29.7	23.3	23.0	2330	1.8	1.9
2005	28.3	22.2	21.5	2296	1.8	1.8
2006	26.4	20.6	19.7	2269	1.8	1.7
2007	25.3	19.8	18.5	2194	1.7	1.6
2008	24.8	19.4	17.9	2220	1.7	1.6
2009	24.2	19.0	17.2	2159	1.7	1.5
2010	23.3	18.2	16.2	2129	1.7	1.4
2011	22.7	17.7	15.6	2166	1.7	1.4
2012	21.3	16.7	14.4	2110	1.7	1.3
2013	20.5	16.1	13.6	2087	1.6	1.2
2014	19.6	15.4	12.8	2100	1.7	1.2
2015	18.3	14.4	11.8	1956	1.5	1.1
2016	17.6	13.9	11.3	1892	1.5	1.0

AAR, age-adjusted rate.

### Trends in the overall incidence and mortality rates

The trend analysis revealed that the age-adjusted trends in incidence and mortality rates have both been decreasing in Japan. Both the incidence and mortality rate decreased significantly throughout the study period. The incidence rate decreased at 10.8% annually from 1999 to 2002, then 5.8% in 2002–2016. Overall, the AAPC in incidence rate was −6.0% (95% confidence interval (95% CI) −6.8 to −5.2) during the entire study period. Similarly, the age-adjusted mortality rate showed a significant decline in the trend, with an APC of 9.9% in 1999–2002, 4.5% in 2002–2014 and 7.8% in 2014–2016. Consequently, the AAPC in overall mortality rate was −5.3% (95% CI −5.9 to −4.6) (Table 2). Although significant decreasing trends were observed, a further rapid annual change is required to accomplish the End TB Strategy.

**Table 2.** Joinpoint analysis of newly notified active tuberculosis incidence and tuberculosis-related mortality rate per 100 000 persons by sex during 1997–2016

	Period 1		Period 2		Period 3		Period 4		Average APC (%), [95% CI]			
	Years	APC (%)	Years	APC (%)	Years	APC (%)	Years	APC (%)	2012–2016		Entire study period	
Incidence												
Total	1997–1999	−0.1	1999–2002	−10.8^a^	2002–2016	−5.8^a^			−5.8^a^	[−6.0 to −5.5]	−6.0^a^	[−6.8 to −5.2]
Male	1997–1999	1.0	1999–2002	−10.8^a^	2002–2016	−6.2^a^			−6.2^a^	[−6.4 to −6.0]	−6.2^a^	[−6.9 to −5.5]
Female	1997–2005	−7.2	2005–2016	−4.6^a^					−4.6^a^	[−5.6 to −3.7]	−5.7^a^	[−6.4 to −5.1]
Mortality												
Total	1997–1999	0.0	1999–2002	−9.9^a^	2002–2014	−4.5^a^	2014–2016	−7.8^a^	−6.2^a^	[−7.8 to −4.5]	−5.3^a^	[−5.9 to −4.6]
Male	1997–1999	0.9	1999–2002	−10.9^a^	2002–2005	−3.3^a^	2005–2016	−5.5^a^	−5.5^a^	[−6.0 to −5.0]	−5.4^a^	[−6.9 to −3.9]
Female	1997–2005	−5.5^a^	2005–2014	−2.5^a^	2002–2016	−9.9^a^			−6.3^a^	[−10.1 to −2.3]	−4.6^a^	[−5.6 to −3.6]

a Significantly different from zero (*p* < 0.05).

APC, annual percentage change; CI, confidence interval.

### Trends in incidence and mortality rates by sex

**Fig. 2.**
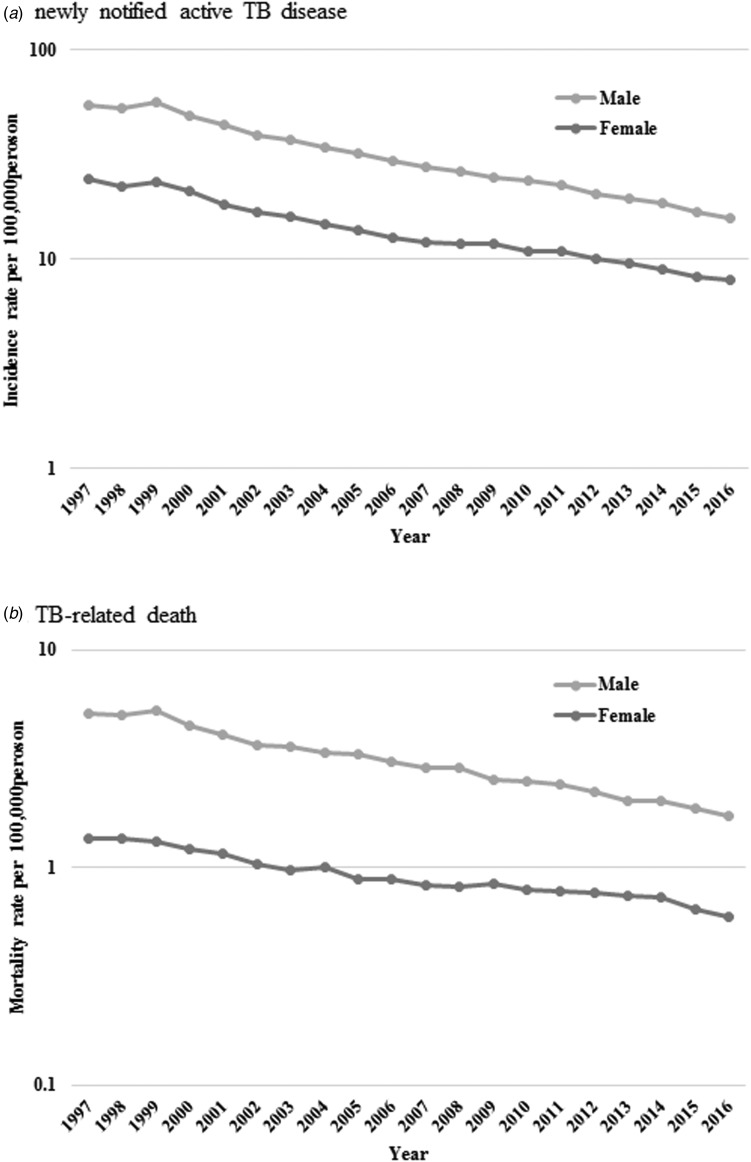
Age-standardised newly notified active tuberculosis incidence (a) and tuberculosis-related death (b) rate per 100 000 persons by sex.

By sex; age-standardised incidence and mortality rate per 100 000 population was determined and the results are shown in Figure 2. Also, the results of the trend analysis are shown in Table 2. The Joinpoint regression analysis revealed that both incidence and mortality rates decreased similarly both in males and females. Incidence rates in males showed an increasing trend between 1997 and 1999, but subsequently decreased. The incidence rate in females continuously declined throughout the study period. Consequently, AAPCs of TB incidence for the entire period in males and females were −6.2% (95% CI −6.9 to −5.5) and −5.7% (95% CI −6.4 to −5.1), respectively. The mortality trend followed a similar chronological course, beginning its rapid decline from 1999 in males and from 1997 in females. AAPC during the current 5-year period showed a slower decline in males (−5.5% (95% CI −6.0 to −5.0)) than females (−6.3% (95% CI −10.1 to −2.3)). For the entire study period, AAPCs in males and females were −5.4% (95% CI −6.9 to −3.9) and −4.6% (95% CI −5.6 to −3.6), respectively.

### Trends in incidence and mortality rates by age

**Fig. 3.**
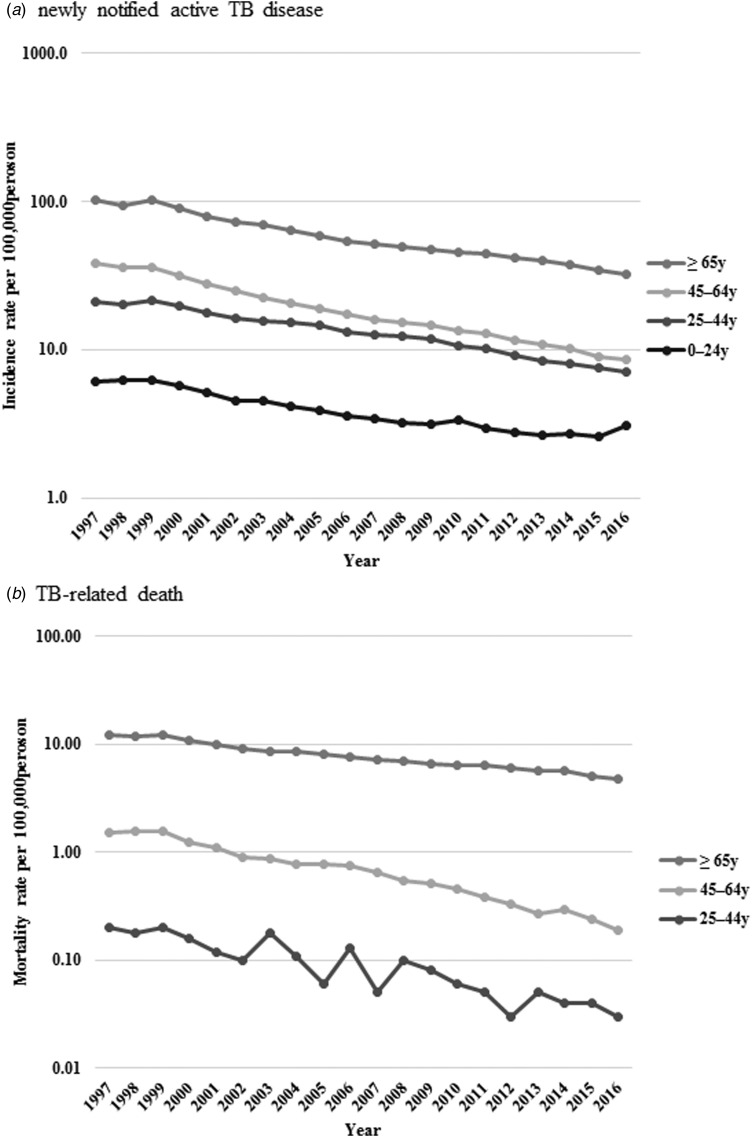
Age-standardised newly notified active tuberculosis incidence (a) and tuberculosis-related death (b) rate per 100 000 persons by age.

Incidence rates per 100 000 population decreased from 6.1 to 3.1, 21.0 to 7.0, 38.7 to 8.6 and 101.4 to 32.2 in the 0–24, 25–44, 45–64 and ⩾65 years age group, respectively. Also, mortality rates per 100 000 population declined from 0.2 to 0.03 among those aged 25–44 years, 1.5 to 0.2 among 45–64 years and 12.0 to 4.8 among ⩾65 years (Fig. 3).

The results of the trend analysis by age group are summarised in Table 3. For the incidence, the Joinpoint regression model did not reveal any remarkable trend in age categories in 1997–1999. Subsequently, there was a change in the trend in 1999, with a decline in the age-adjusted incidence rate towards the end of the study period in all age categories, except the youngest category (0–24 years). The fast-declining trend observed in the youngest age category at 7.7% in 1999–2006 slowed down to 3.6% in 2006–2014, and then stopped decreasing trend in 2014–2016. Also, decreasing trends seen in the middle age group (45–64 years) and older persons (⩾65 years) slowed down at the latter part of the study period. As a result, the AAPC for the entire study period decreased in all age categories; however, the declining trend has decelerated in recent years, except for in the 25–44 years category, and has stopped decreasing in the youngest category.

**Table 3.** Joinpoint analysis of newly notified active tuberculosis incidence and tuberculosis-related mortality rate per 100 000 persons by age during 1997–2016

Age group (years)	Period 1		Period 2		Period 3		Period 4		Average APC (%), [95% CI]			
	Years	APC (%)	Years	APC (%)	Years	APC (%)	Years	APC (%)	2012–2016		Entire study period	
Incidence												
0–24 years	1997–1999	0.0	1999–2006	−7.7^a^	2006–2014	−3.6^a^	2014–2016	6.6	1.3	[−5.9 to 9.1]	−3.8^a^	[−5.7 to −1.8]
25–44 years	1997–1999	1.2	1999–2002	−7.7^a^	2002–2009	−5.0^a^	2009–2016	−7.2^a^	−7.2^a^	[−8.1 to −6.2]	−5.6^a^	[−6.5 to −4.7]
45–64 years	1997–1999	−3.5	1999–2003	−11.4^a^	2003–2016	−6.9^a^			−6.9^a^	[−7.2 to −6.5]	−7.5^a^	[−8.1 to −6.9]
⩾ 65 years	1997–1999	−0.3	1999–2004	−9.0^a^	2004–2016	−5.0^a^			−5.0^a^	[−5.4 to −4.6]	−5.6^a^	[−6.4 to −4.8]
Mortality												
25–44 years	1997–2016	−9.1^a^							−9.1^a^	[−11.2 to −7.0]	−9.1^a^	[−11.2 to −7.0]
45–64 years	1997–1999	0.8	1999–2002	−16.9^a^	2002–2006	−4.5^a^	2006–2016	−12.1^a^	−12.1^a^	[−13.3 to −10.9]	−10.1^a^	[−11.8 to −8.3]
⩾ 65 years	1997–1999	0.1	1999–2002	−9.1^a^	2002–2014	−3.9^a^	2014–2016	−7.4^a^	−5.6^a^	[−8.0 to −3.2]	−4.7^a^	[−5.7 to −3.7]

a Significantly different from zero (*p* < 0.05).

APC, annual percentage change; CI, confidence interval.

On the other hand, mortality rates rapidly and continuously dropped in all age groups in an accelerating manner. APC in the youngest age category (0–24 years) was not applicable due to the limited number of cases. AAPCs for the entire study period showed a significant decrease in every age category: by 9.1% (95% CI −11.2 to −7.0) in 25–44 years, 10.1% (95% CI −11.8 to −8.3) in the middle-aged and by 4.7% (95% CI −5.7 to −3.7) in the oldest age group. Furthermore, AAPCs in the current 5-year period declined more rapidly both in the middle-aged (by 12.1% (95% CI −13.3 to −10.9)) and the oldest (by 5.6% (95% CI −8.0 to −3.2)) age groups.

### Mortality trend by TB category

Finally, we explored mortality trends by TB categories (Table 4). TB cases were divided into three categories: respiratory, nervous system and others. For respiratory TB, there was no significant change in 1997–1999. However, a decline in trend was observed since 1999, with an overall AAPC of −5.5% (95% CI −6.2 to −4.8). Due to few TB cases, APCs for nervous system and the ‘others’ TB categories were determined for the entire study period only; this showed significant declining trends in both categories, −3.0% (95% CI −4.8 to −1.2) and −2.2% (95% CI −2.9 to −1.4), respectively.

**Table 4.** Joinpoint analysis of tuberculosis-related mortality rate per 100 000 persons by tuberculosis category during 1997–2016

	Period 1		Period 2		Period 3		Period 4		Average APC (%), [95% CI]			
	Years	APC (%)	Years	APC (%)	Years	APC (%)	Years	APC (%)	2012–2016		Entire study period	
Respiratory TB	1997–1999	0.5	1999–2002	−10.4^a^	2002–2014	−4.9	2014–2016	−7.6^a^	−6.3^a^	[−8.1 to −4.5]	−5.5^a^	[−6.2 to −4.8]
Nervous system TB	1997–2016	−3.0^a^							−3.0^a^	[−4.8 to −1.2]	−3.0^a^	[−4.8 to −1.2]
Other TB	1997–2016	−2.2^a^							−2.2^a^	[−2.9 to −1.4]	−2.2^a^	[−2.9 to −1.4]

a Significantly different from zero (*p* < 0.05).

APC, annual percentage change; CI, confidence interval.

## Discussion

In the present study, we investigated the newly notified active TB incidence and TB-related mortality rates using the nationwide data. Still moderately endemic, overall TB incidence declined continuously by 6.0% annually during the last two decades, close to the national-level target of 10 per 100 000 population in Japan [4]. However, this decline in the annual incidence of TB diseases in Japan is far from achieving TB elimination by 2035. Although the overall mortality rate decreased by 5.3% annually, it is necessary to further accelerate this declining trend to achieve the End TB Strategy. Although a clear explanation cannot be provided, the downward trends in the AARs for both TB incidence and mortality from the early 2000s was also reported in the global systemic analysis [28]. The global study reported an accelerating decreasing trend in developed countries with high socio-demographic index. Average annualised rate of changes in the age-standardised incidence and mortality rate declined from −1.1% to −3.1% and −1.1% to −7.2%, respectively, between 1990–2005 and 2005–2015 [16]. The decreasing trends in the incidence rates in Japan were faster than the average reported in developed countries, while the mortality trend remained at similar levels [16]. Although the global TB incidence rate is declining in an accelerating manner, there is concern that the declining speed in Japan might be slowing down, especially among the younger population. For global TB elimination, an international study in 33 low-incidence countries presented APC targets for incidence rates as −18% and −11%, on average, by 2035 and 2050, respectively [11]. Thus, greater efforts are required to reduce the TB incidence in Japan towards the global goal of TB elimination.

Compared with men, the Japanese female population had a lower incidence and mortality of TB disease, as was reported from other countries [16,24]. A similar sex difference, associated with TB infection, was described in the global analysis, with 64.7% of deaths in HIV-negative individuals occurring in the male population [28]. Our result of higher TB incidence in the male population indicated that men are more likely to be exposed to TB than similar age women throughout the age categories. Although a conclusive explanation is difficult, men may be more likely to be exposed to TB unknowingly in society. However, as for the risk of latent TB infection, a previous study demonstrated that women are currently at higher risk than men in Japan: 57.5% of notifications for latent TB infection were reported from women [29]. Therefore, we speculate that men, compared with women, have more underlying risk factors for developing TB diseases. Previous literature regarding the characteristics of Japanese active TB cases reported that underlying conditions or morbidity, such as homelessness, HIV-complicated cases and diabetes mellitus, were more frequently observed in men [30]. This difference also possibly accounts for higher mortality in men. As mentioned above, however, the incidence and mortality rates in Japanese males are constantly decreasing. Although a causal explanation is difficult, improvements in treatment compliance and management of underlying diseases may be plausible explanations for the better prognosis of the disease. In addition, TB contact investigations routinely performed by public health centres as a government support could have contributed to the decline of TB prevalence in Japan [31].

Our data demonstrated that there was an annual average TB mortality decrease of approximately 10% in Japanese people under 65 years. Active TB incidence and mortality among older people are still high, though it is steadily decreasing, with an APC of −5.6% and −4.7%, respectively. The older population is globally at greater risk of TB [5,6], with disease burden increases in North and Latin American countries [7,32] and China [33]. In this aged society in Japan, where the proportion of people aged ⩾65 years accounted for 26.7% of the total population in 2015 [34], TB can be a persistent serious public burden over time.

In addition to the incidence, case fatality determines the mortality trend of the disease. The relatively slower decrease in the mortality rate of TB in older people can be explained by several factors. First, underlying chronic diseases in an advanced-age population, including chronic obstructive pulmonary disease, malnutrition and diabetes mellitus, can increase the mortality rate [7,35]. Second, a delay in diagnosis can contribute to a slower decrease in TB mortality among older people [36]. Older adults, particularly those who are physically challenged, are often economically distressed and socially vulnerable, and tend to be discouraged from visiting a medical facility [37]. Also, older people tend to present atypically or with less prominent manifestations of a disease, which can lead to misdiagnosis or delay in diagnosis [7,38]. Furthermore, older people diagnosed with TB may frequently experience adverse effects associated with TB treatment, and they tend to be treated with second- or third-line drugs. Also, in older patients infected with drug-resistant TB, the treatment outcome would be poor as they are vulnerable to prolonged and intensified chemotherapy [39]. As a result of these factors, older adults are at higher risk of developing incurable TB disease and consequent unfavourable prognosis [40], leading to the relatively slower decrease in the mortality rate.

The strength of our study is the nationwide and long-term nature of the data. However, our study had several limitations. First, under-reporting of TB cases should be noted. Diagnostic accuracy of TB diseases might not be perfect in various clinical settings. However, TB diseases are to be reported by law to the national surveillance system in Japan; thus, the diseases are, in general, carefully diagnosed and followed up by clinicians. Second, due to the absence of clinical data, we could not determine whether TB diseases were directly associated with the prognosis of the patients. It is essential to consider HIV co-morbidity, which is often associated with TB mortality. In some European countries, emergence and wide-spread HIV infection altered the rate of decline of TB mortality [41]. Third, our approach to the age adjustment using the European standard population might have yielded a bias, because the demographic structure, as well as the TB epidemiology of Japan, possibly differ. However, compared with the rest of the world, European countries have similar social and population structures (industrialised countries with ageing populations), and thus our age-adjustment method would be comparably suitable. Our principle aim of the study was to evaluate the trend of TB diseases in Japan, and the present method would satisfy this purpose. Finally, long-term case fatality should have been traced to estimate the trend change of TB diseases impacts. However, due to a lack of relevant data available, we could not directly evaluate this in this study.

In conclusion, we revealed the long-term trends in TB incidence and mortality rates in Japan, which have certainly declined over the past two decades. Mortality, especially among the young to middle-aged population, is favourably decreasing, by approximately 10% annually. On the other hand, the decline in the overall incidence rate (annual APC, −6.0%) goes below the target goal of the End TB Strategy, which is a potential reason that Japan is a moderately TB endemic country. Further multisectoral measures based on a long-term epidemiological assessment are necessary.
